# Investigation of *VASA* Gene and Protein Expression in Neonate and Adult Testicular Germ Cells in Mice *In Vivo* and *In Vitro*

**DOI:** 10.22074/cellj.2020.6619

**Published:** 2019-10-14

**Authors:** Hossein Azizi, Mojtaba Ranjbar, Somayeh Rahaiee, Mostafa Govahi, Thomas Skutella

**Affiliations:** 1 Faculty of Biotechnology, Amol University of Special Modern Technologies, Amol, Iran; 2 Institute for Anatomy and Cell Biology, Medical Faculty, University of Heidelberg, Im Neuenheimer Feld, Heidelberg, Germany

**Keywords:** Germ Cells, Mouse, Spermatogonial Stem Cells, Testis, VASA

## Abstract

**Objective:**

We aimed to examine the expression levels of the *VASA* gene and protein in testis sections of neonate and adult mice as well as testicular cell cultures.

**Materials and Methods:**

In this experimental study, in order to investigate the expression of this germ cell marker gene in more detail, we analyzed the expression of VASA by immunocytochemistry, immunohistochemistry and fluidigm reverse transcription-polymerase chain reaction (RT-PCR).

**Results:**

The immunohistochemical assays showed that the VASA protein was exclusively expressed in germ cells in the seminiferous tubules of the neonate and adult testis and not in somatic cells. VASA was not detectable in PLZF positive spermatogonial stem cells (SSCs), was weakly expressed in proliferating spermatogonia, and became abundant in spermatocytes and round spermatozoa. Counting VASA-positive cells in the seminiferous tubules of the neonate and adult testis depicted significant higher expression (P<0.05) of VASA in the adult testis in comparison to its neonate counterpart. SSC colonies were established *in vitro* after digestion of the testis and characterized by immunocytochemistry for CD90 and stage-specific embryonic antigens 3 (SSEA3). Immunocytochemistry confirmed that in contrast to the not detectable signal *in vivo*, VASA protein was strongly localized in the cytoplasm of both neonate and adult mouse SSCs under *in vitro* conditions. The results of Fluidigm RT-PCR revealed a significant higher expression of the germ cell gene VASA in adult SSCs in comparison to neonate SSCs in cell culture (P<0.05).

**Conclusion:**

The VASA protein is, therefore, an extremely specific marker of testicular germ cell differentiation *in vivo* and mostly expressed in the adult testis in spermatocytes and round spermatids. The immunohistochemical signal in spermatogonia is very low. So, PLZF positive SSCs are negative for VASA *in vivo*, while in contrast, once isolated from the testicular niche VASA is also strongly expressed in SSCs under *in vitro* conditions.

## Introduction

In most animal species germ cells go through two complex stages of development. In the first stage, which takes place throughout early embryogenesis, primordial germ cells are generated and actively migrate to the gonadal anlage, which is supposed to comprise all of the somatic components necessary to establish the mature gonads (1, 2). During the second stage of germline development, the germ cells are provided with appropriate cues from the gonadal somatic environment and recruit one of two separate developmental programs of either oogenesis or spermatogenesis to form sexspecific gametes (1, 3). Spermatogonial stem cells (SSCs) are the unipotent adult stem cells of the testis that participate in spermatogenesis and can proliferate under certain cell culture conditions (4, 5). The amount of undifferentiated Oct4- positive SSCs in the adult mouse testis section is very low (4). In the germline epithelium, SSCs are located directly in the stem cell compartment above the basement membrane of the seminiferous tubules enclosed by Sertoli cells. Patches of Leydig cell islands, blood vessels, and macrophages are localized in the peritubular space. These kinds of somatic cells in combination with the peritubular fibrocytes and intratubular Sertoli cells secrete factors that regulate the self-renewal and differentiation of SSCs (6). In the testicular niche, extrinsic and intrinsic factors regulate the maintenance of SSCs. At least some extrinsic factors, such as glial cellderived neurotrophic factor (GDNF) (4, 7) and KIT ligand (KITL) (8), are produced and delivered by Sertoli cells. Intrinsic factors-genes essential for regulating the mitotic phase of spermatogenesis - include transcription regulators such as the Zinc finger and the BTB domain containing 16 (ZBTB16/PLZF) (7, 9), ETS variant gene 5 (ETV/ERM) (10), Taf4b (11), Atm (12); Bcl6b (13); Pin1 (14), Pou5f1, Nrg1, Nanog and Gja1 (15) and the GDNF receptor complex Gfra1, RET (16).

The VASA gene has been firstly found to be essential for the development of female germ stem cells (GSCs) in Drosophila (17). In mice with systematic genetic deletions of the VASA gene, males exhibit a reproductive deficiency with a loss of sperm production. The male GSCs die at the zygotene step of meiosis phases, whereas the ovarian function appears to be normal (18). It has been observed that VASA is localized in PGCs in mice from embryonic day 12.5 onwards directly after entering the gonadal anlage (18-20).

In the current study, we have extended our research on the expression of VASA in the neonate and adult testis sections and testicular culture to determine if VASA has the same pattern of expression in testicular germ cells both *in vivo* and *in vitro*.

## Materials and Methods

### Tissue digestion and culture of testicular cells

In this experimental study, animal experiments were approved by Amol University of Special Modern Technologies Ethical Committee (Ir.ausmt.rec.1398.03.07). Testis cells from 6 day- to 12 week-old mice from the C57BL/6 strain were isolated by a one-step enzymatic digestion solution, which contained collagenase IV (0.5 mg/ml, Sigma Aldrich, USA), DNAse (0.5 mg/ml, Sigma Aldrich, USA) and Dispase (0.5 mg/ml, Sigma Aldrich, USA) in a Hank’s Balanced Salt Solution (HBSS) buffer with Ca++ and Mg++ (PAA, USA) (21). The suspension of digested testis cells was plated in SSCs medium, which contained StemPro-34 medium, 1% N2-supplement (Invitrogen, USA), 6 mg/ml D+ glucose (Sigma Aldrich, USA), 5 μg/ml bovine serum albumine (Sigma Aldrich, USA), 1% L-glutamine (PAA, USA), 0,1% ß-mercaptoethanol (Invitrogen, USA), 1% penicillin/streptomycin (PAA, USA), 1% MEM vitamins (PAA, USA), 1% non-essential amino acids (PAA, USA), 30 ng/ml estradiol (Sigma Aldrich, USA), 60 ng/ml progesterone (Sigma Aldrich, USA), 20 ng/ml epidermal growth factor (EGF, Sigma Aldrich, USA), 10 ng/ml fibroblast growth factor (FGF, Sigma Aldrich, USA), 8 ng/ml GDNF (Sigma Aldrich, USA), 100 U/ml human leukemia inhibitory factor (LIF, Millipore, USA), 1% ES cell qualified FBS, 100 μg/ml ascorbic acid (Sigma Aldrich, USA), 30 μg/ml pyruvic acid (Sigma Aldrich, USA) and 1 μl/ml DL-lactic acid (Sigma Aldrich, USA) at 37˚C and 5% CO_2_(4).

### Gene expression analyses on the Fluidigm Biomark system

Measurements of the expression of the gene DEAD (Asp-Glu-Ala-Asp) box polypeptide 4 (DDX4 or VASA) Mm00802445_m1 in the neonate and adult SSCs were analyzed with Dynamic Array chips (Fluidigm). A housekeeping gene glyceraldehyde-3-phosphate dehydrogenase (*GAPDH*) Mm99999915_g1 was used for normalization, in different types of cultured cells. SSCs were picked with a micromanipulator, lysed with a lysis buffer solution containing 9 μl RT-PreAmp Master Mix (5.0 μl Cells Direct 2× Reaction Mix) (Invitrogen, USA), 2.5 μl 0.2× assay pool, 0.2 μl RT/Taq Superscript III (Invitrogen, USA) and 1.3 μl TE buffer. The number of RNAtargeted transcripts was measured using TaqMan PCR assays on the BioMark Real-Time quantitative PCR (qPCR) system. Each sample was analyzed in two technical repeats. The Ct values were examined using GenEx software from MultiD for analysis (4, 5).

### Immunocytochemical staining

In this experimental study, samples were fixed with 4% paraformaldehyde (PFA)/phosphate buffered saline (PBS) and permeabilized with 0.1% Triton/PBS solution. Samples were blocked with 1% bovine serum albumin (BSA)/PBS buffer and incubated overnight with the primary antibody against VASA (Abcam, USA), PLZF (Millipore, USA), CD90 (Abcam, USA) and SSEA3 (R<D, USA). This step was followed by incubation with secondary antibodies. We diluted the primary and secondary antibodies at 1: 200. The labeled cells were counterstained with 0.2 μg/ml DAPI (4’, 6-diamidino-2-phenylindole) (Sigma, USA). Fluorochrome positive cells were studied with a Zeiss LSM 700 confocal microscope (Zeiss, Germany), and images were acquired with a Zeiss LSM-TPMT camera (Zeiss, Germany) (4, 22).

### Tissue processing for immunohistofluorescence staining

Testis tissue samples were selected from neonate and adult male animals, washed twice in PBS buffer, and fixed in 4% PFA. The testis tissue samples were dehydrated and embedded in Paraplast Plus. The tissue samples were then cut with a microtome at a thickness of approximately 10 μm. The tissue sections were mounted on super frost plus slides and kept at room temperature. Before staining, sections were deparaffinized in xylene and rehydrated in a graded ethanol series. Following heat induced epitope retrieval (using a microwave), non-specific binding of antibodies and other detection agents were blocked with 10% serum/0.3% Triton in PBS, and ICC staining continued as described above (4).

### Statistical analysis

The experiments were replicated at least three times. The average number of VASA-positive cells in groups were evaluated using the one-way analysis of variance (ANOVA), followed by Tukey’s posthoc test. The expression of VASA was compared with non-parametric Mann-Whitney’s test. The variation between neonate and adult groups was considered statistically reliable if a value of P<0.05 had been acquired. All statistical testes were performed using Statistical Package for the Social Sciences (SPSS) softwere.

## Results

**Fig 1:**
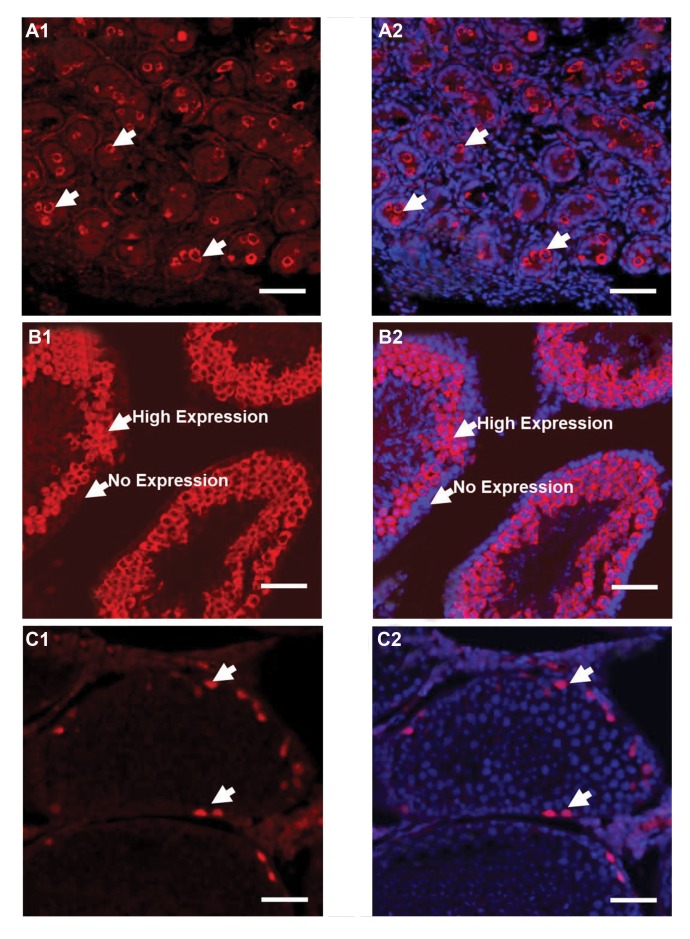
Immunocytochemical characterization in testis sections. **A.** Immunohistochemistry for the expression of VASA in the cross-section of the neonate testis, **A1.** Red fluorescence for VASA, A2. Merged image for red fluorescence for VASA and blue fluorescence for DAPI, **B.** Immunohistochemistry for the expression of VASA in the cross-section of the Adult testis, **B1.** Red fluorescence for VASA, **B2.** Merged image for red fluorescence for VASA and blue fluorescence for DAPI, **C.** PLZF protein was expressed in the base compartment of the seminiferous tubules of the testis, **C1.** Red fluorescence for PLZF, and **C2.** Merged image for red fluorescence for PLZF and blue fluorescence for DAPI. The arrows show the expression of related protein in the seminiferous tubule.

**Fig 2:**
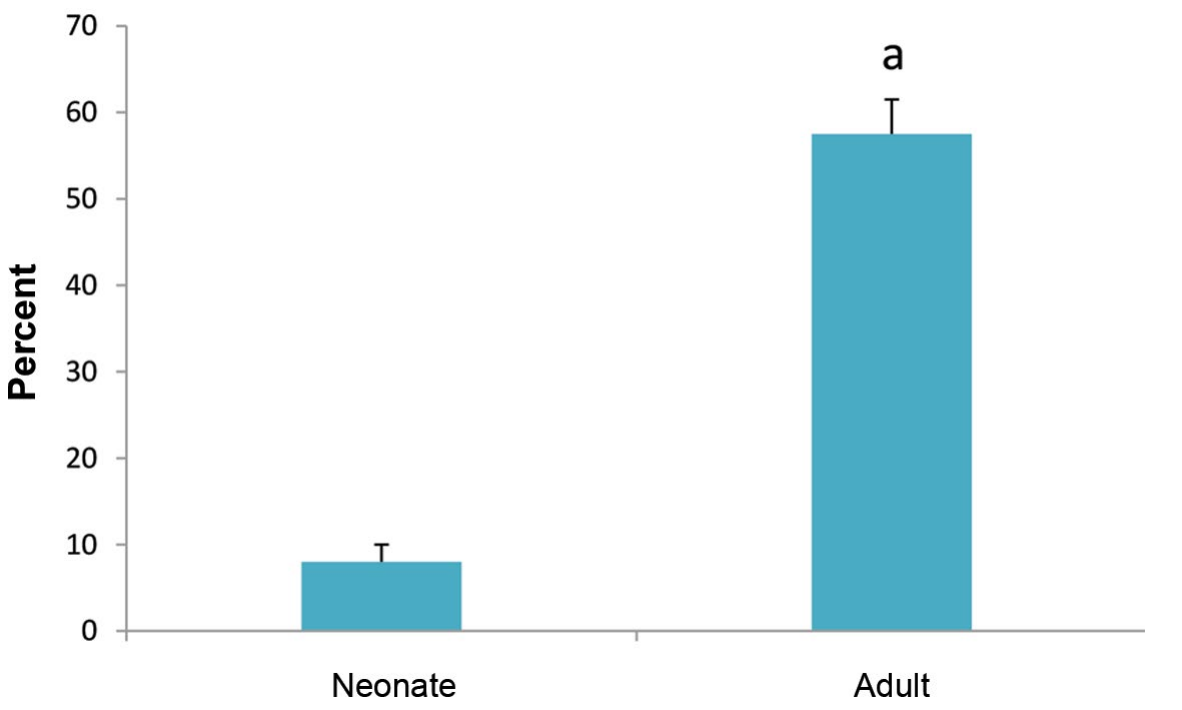
Number of VASA-positive cells in testis sections. Counting of VASA-positive cells in the testis section of the neonate and adult mouse. a; At least P<0.05 versus other groups.

**Fig 3:**
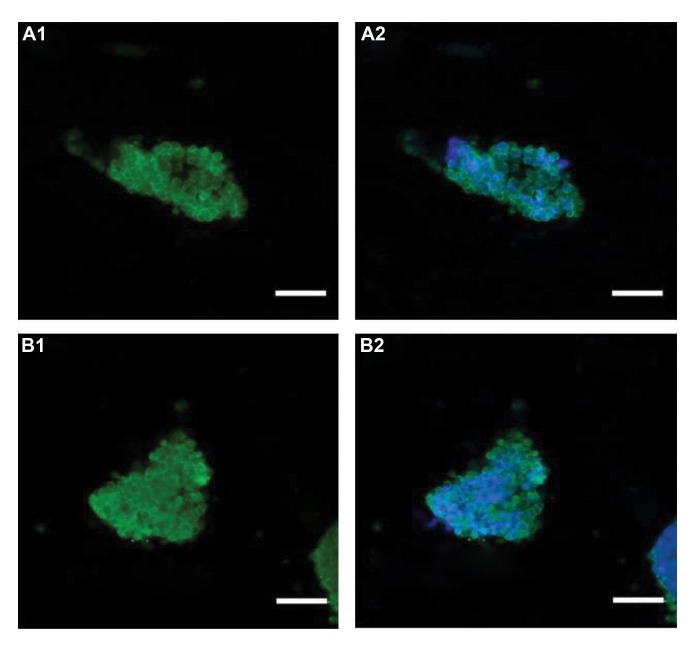
Characterization of spermatogonial stem cells (SSCs). **A.** Immunocytochemistry of generated SSCs with CD90 and **B.** SSEA3 antibodies. **A1.** Green fluorescence for CD90, **A2.** Merged image for green fluorescence for VASA and blue fluorescence for DAPI, **B1.** Green fluorescence for SSEA3, and **B2.** Merged image for green fluorescence for SSEA3 and blue fluorescence for DAPI.

**Fig 4:**
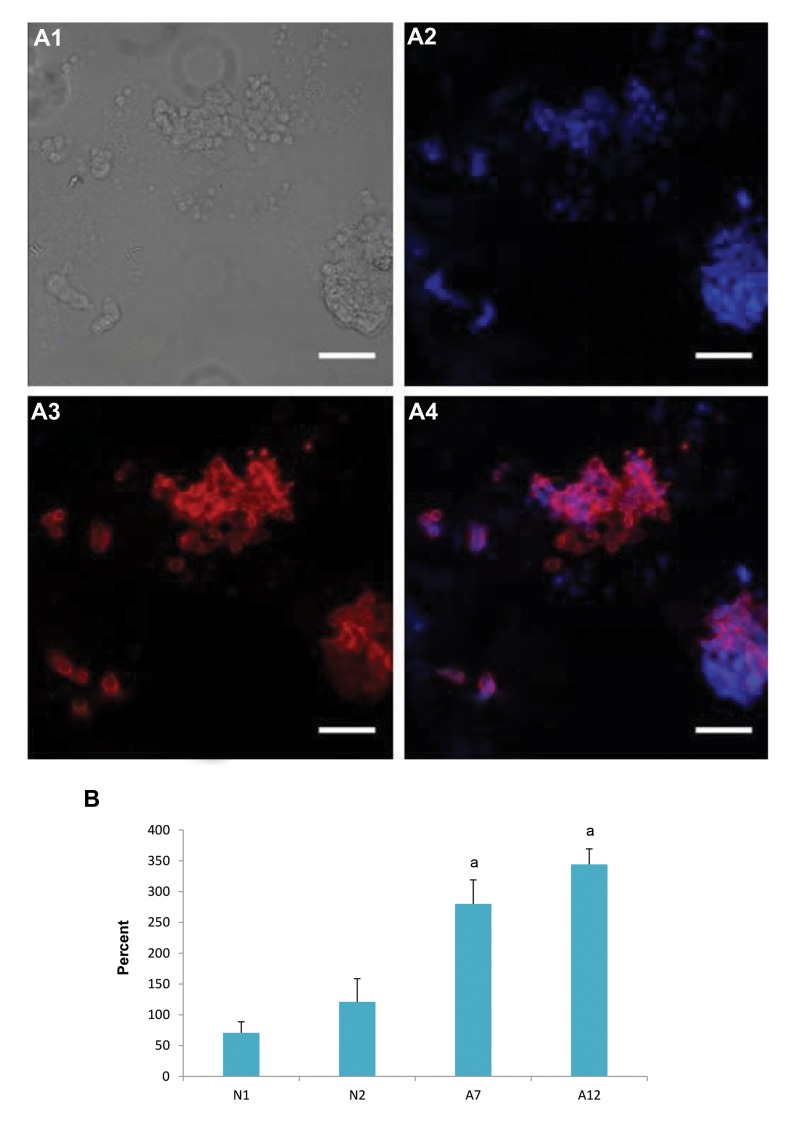
Immunocytochemistry and quantitative polymerase chain reaction (PCR) for spermatogonial stem cells (SSCs). **A.** Immunocytochemistry for generated SSCs using anti-VASA antibody. **A1.** Bright field photo of SSC colony, **A2.** DAPI staining which shows all cells in the plate, **A3.** VASApositive cells, **A4.** Merge, and **B.** Quantitative PCR analysis for the expression of VASA in the neonate and adult testis. a; At least P<0.05 versus other groups.

In the first step, we examined the expression of VASA in the neonate (Fig .1A1, Fig A2) and adult testis (Fig .1B1, Fig B2) through immunohistochemistry. Immunohistochemistry with confocal microscopy revealed that the VASA protein was expressed in the seminiferous tubules of both the neonate and adult testis but with different localizations. In the neonate testis, VASA-positive cells were located in the center of the seminiferous tubules, while in the adult testis sections, the cells were distributed through spermatogonia, spermatocytes, and spermatids with the exclusion of SSCs located in the cell layer directly connected to the base membrane of the seminiferous tubule and were also abundant in sperm. The staining of the spermatogonia, directly in contact with the basement membrane, was weak while the PLZF protein was clearly expressed in this region (Fig.1(. Counting of VASApositive cells in the seminiferous tubules of the neonate and adult testis showed that about 8% of the cells in the neonate and 57% of the cells in the adult testis were positive. Therefore, a higher number of VASA-positive cells were observed in the adult testis (Fig .2. P<0.05). In the next step, we evaluated the expression level of VASA in neonate and adult SSCs. Analysis of immunocytochemistry images revealed that the generated SSCs were positive for the CD90 and SSEA3 markers (Fig .3). Quantitative PCR analysis using single cells revealed that the expression of VASA mRNA in adult SSCs was significantly higher than in neonate SSCs (P<0.05, expression fold change of VASA mRNA analyzed on MEF feeder cells). Following immunocytochemistry, we observed no difference in the expression of VASA at the protein level in neonate and adult SSCs (Fig .4). The characterization of SSCs was conducted as designated in our former study (4).

## Discussion

In the seminiferous tubules of the neonate mouse, the expression of VASA was specifically expressed in the center of the testicular cords. It seems that these cells were T1-prospermatogonia. During the first postnatal week, the T1-prospermatogonia relocate to the seminiferous tubules and form T2 prospermatogonia. These cells start to populate the basement membrane and initiate the spermatogenesis pathway throughout post-pubertal life (23). We observed the expression of the VASA protein in spermatocytes located above the spermatogonial cell layer in the seminiferous tubule of the adult mouse testis, and a decrease of VASA protein expression during spermiogenesis. Our study demonstrated that the expression of VASA was evident in the spermatocytes and round spermatids of the adult mouse testis, absent in SCCs and weakly expressed in spermatogonia in neonate and adult mouse testis sections. We also confirmed that the expression of VASA in the adult mouse SSCs was higher than in neonates with Fluidigm RT-PCR.

Our experiment demonstrated that SSCs generated under the stimulation of the growth factors FGF, EGF and GDNF expressed CD90 and SSEA3 (24). We observed that in contrast to the *in vivo* situation, SSCs in culture express higher amounts of VASA. This might be due to histological changes in the stem cell compartment, including feeders and the separation from Sertoli cells. In mice, the preservation and amount of male germ line stem cells *in vitro* could be diminished by somatic Sertoli feeder cells (25). Zebra fish SSCs are also differentiated into functional sperm under the effect of these feeder cells in culture (26). In contrast, some studies have reported of the suitability of Sertoli cell feeder layers for long term *in vitro* culture of SSCs (27, 28). Bovine fetal fibroblasts have been shown to promote maintenance of bovine undifferentiated spermatogonia for at least two months (29). It has also been demonstrated that amniotic epithelial cells retain SSCs, which are capable of self-renewal, in an undifferentiated, proliferative state (30).

It has been reported that bFGF is an efficient growth factor for the *in vitro* proliferation of primordial germ cells (31, 32). Furthermore, it has been shown that bFGF may be an important factor in addition to GDNF and GFR-α1 for inducing SSC replication on Sertoli cell feeders (4, 27). Also, there are recent studies on human testes that imply a critical function of bFGF in SSC proliferation (5, 33). In contrast, Kuijk et al. (34) observed that FGF can impede the successful derivation of porcine SSCs from the neonate pig testis. This is while it has been demonstrated that TGFß1, insulin-like growth factor I and FGF promoted the cell proliferation of goat SSCs (35). Additionally, the growth conditions generally used for mouse SSCs have been shown to be insufficient for the proliferation of human SSCs (36).

Our results are in line with previous studies, suggesting that VASA is a germline marker during spermatogenesis and also in proliferating spermatogonia (19, 37). We observed that VASA expression is not detectable in PLZF-positive SCCs of the mouse testis. Choi et al. (38) demonstrated that SSCs from Oct4 reporter mice cultured under feeder-free conditions expressed the SSC marker genes *Oct4* and *Vasa*, which is in accordance with our observation of VASA-positive SSCs under *in vitro* conditions. Further studies are required to analyze the possible mechanisms involved in the regulation of VASA expression during SSC self-renewal *in vivo* in comparison to *in vitro* and the influence exerted by Sertoli cells.

## Conclusion

Our findings indicate that VASA is expressed in both the neonate and adult testis and also in testicular cultures, the rate of expression in the adult testis was higher than in its neonate counterpart. In the future it would be of interest to understand why VASA is not expressed in the PLZF-positive SSCs of the adult mouse testis whereas it is highly expressed in SSCs *in vitro*.
